# The *CXCL12* SNPs and their haplotypes are associated with serum lipid traits

**DOI:** 10.1038/s41598-019-55725-3

**Published:** 2019-12-20

**Authors:** Ling Qiu, Rui-Xing Yin, Rong-Jun Nie, Xi-Jiang Hu, Eksavang Khounphinith, Fen-Han Zhang

**Affiliations:** 1 Department of Cardiology, Institute of Cardiovascular Diseases, The First Affiliated Hospital, Guangxi Medical University, Nanning, 530021 Guangxi People’s Republic of China; 2 Guangxi Key Laboratory Base of Precision Medicine in Cardio-cerebrovascular Disease Control and Prevention, Nanning, 530021 Guangxi People’s Republic of China; 3 Guangxi Clinical Research Center for Cardio-cerebrovascular Diseases, Nanning, 530021 Guangxi People’s Republic of China

**Keywords:** Dyslipidaemias, Dyslipidaemias, Cardiovascular genetics, Cardiovascular genetics

## Abstract

The relationship among the single nucleotide polymorphisms (SNPs) of the C-X-C motif chemokine ligand 12 gene (*CXCL12*) and the serum lipid profiles in the Chinese population has rarely been described, especially in somewhat old-fashioned and isolated Maonan minority. The goal of the current study was to elucidate the connection among the *CXCL12* rs501120 and rs1746048 SNPs, haplotypes, several environmental factors and serum lipid traits in the Maonan as well as Han populations. Genotyping of the two SNPs, gel electrophoresis and direct sequencing were accomplished in 1,494 distinct subjects (Maonan, 750 and Han, 744) using polymerase chain reaction and restriction fragment length polymorphism. The frequencies of genotypes as well as alleles of the two SNPs were not similar between the two ethnic groups. The rs501120 SNP was related with serum total cholesterol levels, while the rs1746048 SNP was related with serum apolipoprotein (Apo) B levels. Four haplotypes were identified, of which the rs501120A-rs1746048C haplotype was the most common. The haplotypes of rs501120A-rs1746048T increased and rs501120G-rs1746048C decreased the risk of hyperlipidemia (*P* < 0.001 for each), showing consistent association with the levels of serum triglyceride, ApoA1 and ApoB. These outcomes specify that the *CXCL12* SNPs as well as their haplotypes are related to serum lipid levels. Different serum lipid levels between both populations may partially be related to the *CXCL12* SNPs, their haplotypes along with several environmental factors.

## Introduction

Recent reports have found that cardiovascular disease (CVD) gives rise to high morbidity, disability and mortality around the world^1^. The death from coronary artery disease (CAD) has increased about 35% over the 20 years from 1990 to 2010, and CAD has become one of the primary causes to mortality^2^. It is extensively accepted that dyslipidemia is strongly related to the risk of CAD^3^. To acquire the risk evaluation of CAD, we conducted a survey about standard lipid profiles, involving serum levels of total cholesterol (TC)^4^, triglyceride (TG)^5^, high-density lipoprotein cholesterol (HDL-C)^6^, low-density lipoprotein cholesterol (LDL-C)^7^, and apolipoprotein (Apo) A1, ApoB and the ratio of ApoA1 to ApoB^6^ in the Maonan and Han populations.

Until now, genome-wide association studies (GWASes) have recognized abundant genetic mutations related to the risk of CAD^8^, and various environmental factors with their interactions could have an impact on serum lipid levels^9^, such as gender, age, obesity, cigarette smoking, physical inactivity, diabetes, and hypertension^10–14^. When it comes to genetic factor, we found one gene and two novel single nucleotide polymorphisms (SNPs) which were reported from previous GWASes and other genetic studies^8,15–17^. The C-X-C motif chemokine ligand 12 (CXCL12, or called stromal cell-derived factor 1, SDF1) is encoded by the *CXCL12* on chromosome 10. This gene is an important member of the CXC subfamily of chemokines that is generally expressed in several human tissues and cell types^18^. The *CXCL12* includes one of 27 SNPs related with augmented risk of CAD^19^, and two adjacent SNPs of rs501120 and rs1746048 were also related with CAD^15,17^.

In 2007 Samani *et al*. reported that the rs501120 SNP was suggestively related with CAD^15^. In 2009, they reported again that the rs501120 SNP had a possibility of gender interaction on CAD according to their large-scale analysis of genetic loci for CAD^20^. In the meanwhile, a progress report also discussed the connotation among the rs501120 locus and genetic risk prediction of myocardial infarction^21^. Ansari *et al*. found that the rs1746048 SNP influenced the levels of cytokine (IL-18 and the ratio of IL-18/IL-10) in premature CAD patients, and it could result in cytokine imbalance^22^. Besides, it has been also documented that the rs501120 SNP was related to heart failure and associated with TC, LDL-C as well as ApoB levels in CAD patients^23^, and the rs1746048 SNP was related with LDL-C concentrations in some dyslipidemic patients^24^. Moreover, Sivapalaratnam *et al*. reported a putative mechanism about these two SNPs that they were associated with neointima development following arterial injury, together with platelet stimulation in atherosclerotic lesions^25^. Although the two SNPs located in downstream of *CXCL12* are non-coding DNA, they may be transliterated into efficient non-coding RNA molecules. Recent studies suggested that regulatory non-coding RNAs have an imperative function in epigenetic control^26^. In addition, some non-coding DNAs are also involved in epigenetic regulations and associated with the development of complex organisms^27^. There will be interactions between SNPs and potential epigenetic changes in gene function as we hypothesized. However, it is still unclear whether the *CXCL12* rs501120 and rs1746048 SNPs are related to serum lipid concentrations, or if they demonstrate any ethnic and/or gender specific associations.

China has 56 diverse ethnic groups. Han is the biggest group and Maonan is one of 55 minorities with a population of 101,192^28^. The Maonan ethnic group is mostly allocated in Shangnan, Zhongnan as well as Xia’nan towns of Huanjiang Maonan Autonomous County in Southwestern China. The Maonan group has a small population, but it’s well recognized throughout China for its unique history as well as culture. The unique customs along with precise characteristics, such as clothing, ethnic marriages, eating habits as well as lifestyle factors of Maonan ethnic group differ from those of Han people who live nearby^29^. They are mainly engaged in agricultural production, supplemented by animal husbandry: being good at raising beef cattle. They have their own dietary habits: the main food is rice, but corn, sorghum, millet, sweet potatoes as well as pumpkins are also imperative dietary supplements, and they are fond of spicy and acid or sour foods. Therefore, they have a precise distinctive lifestyle as well as dietary practices compared with other ethnic groups. Their elders arranged the marriages of Maonan descendants. Maonan maintains endogamy, namely marriage with Han or other minorities, like Zhuang in China, is rare, so it is considered that the genetic background of Maonan community might have low population heterogeneity. The inherited features along with genotypes of specific genes related to lipid metabolism in Maonan population may differ from the ones in the Han. Hence, the goal of the present study was to investigate the possible relationship of the *CXCL12* rs501120 and rs1746048 SNPs plus some environmental exposure with serum lipid concentrations among the populations of Maonan and Han.

## Results

### General features along with serum lipid profiles

As mentioned in Table 1, body weight (wt.), waist circumference, systolic blood pressure (SBP), diastolic blood pressure (DBP), the levels of serum TG, and ApoB were greater in Maonan compared to Han (*P* < 0.05), while the concentrations of serum HDL-C were lesser in Maonan than in Han (*P* < 0.001). There was not any substantial alteration in sex ratio, age, height, body mass index (BMI), the fraction of participants who drank alcohol or smoked cigarettes, the concentrations of blood glucose, TC, LDL-C and ApoA1, and the ratio of ApoA1 to ApoB (ApoA1/ApoB) (*P* > 0.05 for all).

**Table 1:** Comparison of demographic, lifestyle characteristics and serum lipid levels between the Maonan and Han populations

Parameter	Maonan	Han	t (χ^2^)	P
Number	750	744		
Gender (male/female)	324/426	270/474	1.143	0.256
Age (years)^a^	55.71 ± 15.10	54.08 ± 15.49	1.476	0.132
Height (cm)	154.81 ± 7.99	153.72 ± 7.73	1.904	0.057
Weight (kg)	54.56 ± 10.55	52.70 ± 8.74	2.618	0.009
Body mass index (kg/m^2^)	22.62 ± 3.14	22.29 ± 3.37	1.377	0.169
Waist circumference (cm)	77.69 ± 9.40	74.83 ± 7.77	6.467	1.365E-10
**Cigarette smoking [n (%)]**				
Non-smoker	572(76.27)	562(75.54)		
≤20 cigarettes per day	148(19.73)	160(21.51)	1.762	0.414
>20 cigarettes per day	30(4.00)	22(2.95)		
**Alcohol consumption [n (%)]**				
Non-drinker	582(77.60)	608(81.72)		
≤25 g per day	86(11.46)	66(8.87)	4.123	0.127
>25 g per day	82(10.94)	70(9.41)		
Systolic blood pressure (mmHg)	137.28 ± 24.91	129.52 ± 20.04	6.655	4.033E-11
Diastolic blood pressure (mmHg)	83.80 ± 13.73	81.33 ± 12.10	2.608	0.009
Pulse pressure (mmHg)	53.49 ± 18.66	48.19 ± 15.97	5.840	6.439E-9
Glucose(mmol/L)	6.12 ± 1.28	5.99 ± 1.56	1.200	0.231
Total cholesterol (mmol/L)	4.99 ± 1.05	4.95 ± 1.01	0.447	0.655
Triglyceride (mmol/L)^b^	1.28(0.93)	1.03(0.81)	2.188	0.029
HDL-C (mmol/L)	1.59 ± 0.41	1.76 ± 0.53	−7.035	3.031E-12
LDL-C (mmol/L)	2.90 ± 0.86	2.80 ± 0.81	1.701	0.089
Apolipoprotein (Apo) A1 (g/L)	1.35 ± 0.26	1.39 ± 0.37	−1.671	0.095
ApoB (g/L)	0.88 ± 0.20	0.85 ± 0.20	2.405	0.016
ApoA1/ApoB	1.67 ± 0.64	1.68 ± 0.51	−0.247	0.805

HDL-C, high-density lipoprotein cholesterol; LDL-C, low-density lipoprotein cholesterol. ^a^The quantitative variables were presented as mean ± standard deviation and determined by Student’s unpaired *t*-test. ^b^The value of triglyceride was presented as median (interquartile range) for not meet the normal distribution, the difference between the two ethnic groups was determined by the Wilcoxon-Mann-Whitney test.

### Outcomes of electrophoresis as well as genotyping

After amplification of the genomic DNA by polymerase chain reaction (PCR) and observation of the *CXCL12* rs501120 SNP by 2% agarose gel electrophoresis, the PCR product of 521 bp nucleotide sequences was acquired (Supplementary Fig. 1A). The PCR product of the *CXCL12* rs1746048 SNP was 257 bp (Supplementary Fig. 1B). The *CXCL12* rs501120 AA (521 bp), AG (521-, 293- and 230-bp) as well as GG (293- and 230-bp) genotypes are presented in the Supplementary Fig. 2A. The *CXCL12* rs1746048 TT (257 bp), CT (257-, 218- and 39-bp) as well as CC (218- and 39-bp) genotypes are presented in Supplementary Fig. 2B. The genotypes of the two SNPs distinguished by polymerase chain reaction and fragment length polymorphism (PCR-RFLP) were also established by direct sequencing (Supplementary Fig. 3).

### Genotypic and allelic frequencies

The genotypic as well as allelic frequencies of the rs501120 SNP were dissimilar in both groups. The occurrences of the rs501120G allele (29.7% *vs*. 34.9%) and rs501120GG genotype (7.5% *vs*. 10.3%) were lesser in Maonan than in Han. The genotypic as well as allelic frequencies of the rs1746048 SNP in both populations were also dissimilar (*P* < 0.05 for each). The genotypic dispersal of both loci was steady with the Hardy-Weinberg equilibrium. Subgroup evaluation indicated that there was not substantial alteration in the genotypic as well as allelic frequencies between men and women in two ethnic groups (Table 2).

**Table 2:** Comparison of the *CXCL12* rs501120 and rs1746048 genotypic and allelic frequencies between the Maonan and Han populations.

SNP/Group	*n*	Genotype [*n* (%)]			Allele [*n* (%)]		*P*_HWE_
		AA	AB	BB	A	B	
*CXCL12* rs501120							
Maonan	750	362(48.3)	331(44.2)	57(7.5)	1055(70.3)	445(29.7)	0.115
Han	744	316(42.5)	336(45.2)	92(10.3)	968(65.1)	520(34.9)	0.854
χ^2^	—	11.356			9.522		
*P*	—	0.003			0.002		
Maonan							
Male	326	156(47.9)	147(45.1)	23(7.1)	459(70.4)	193(29.6)	0.139
Female	424	206(48.6)	184(43.4)	34(8.0)	596(70.3)	252(29.7)	0.423
χ^2^	—	0.366			0.002		
*P*	—	0.833			0.961		
Han							
Male	271	111(41.0)	132(48.7)	28(10.3)	354(65.3)	188(34.7)	0.217
Female	473	205(43.4)	204(43.1)	64(13.5)	614(64.9)	332(35.1)	0.246
χ^2^	—	2.843			0.025		
*P*	—	0.241			0.874		
*CXCL12* rs1746048							
Maonan	750	369(49.2)	324(43.2)	57(7.6)	1062(70.8)	438(29.2)	0.220
Han	744	333(44.8)	325(43.7)	86(11.5)	991(66.6)	497(33.4)	0.621
χ^2^	—	7.705			6.130		
*P*	—	0.021			0.013		
Maonan							
Male	323	152(47.1)	143(44.2)	28(8.7)	447(69.2)	199(30.8)	0.489
Female	427	217(50.8)	181(42.4)	29(6.8)	615(72.0)	239(28.0)	0.286
χ^2^	—	1.532			1.414		
*P*	—	0.465			0.234		
Han							
Male	272	113(41.5)	128(47.1)	31(11.4)	354(65.1)	190(34.9)	0.561
Female	472	220(46.6)	197(41.7)	55(11.7)	637(67.5)	307(32.5)	0.286
χ^2^	—	2.118			0.898		
*P*	—	0.347			0.343		

Allele A, rs501120A and rs1746048T; Allele B, rs501120G and rs1746048C; Genotype AA, rs501120AA and rs1746048TT; Genotype AB, rs501120AG and rs1746048TC; Genotype BB, rs501120GG and rs1746048CC. HWE, Hardy-Weinberg equilibrium. The genotype distribution between the two groups was analyzed by the chi-square test. The HWE was analyzed by the chi-square test of the goodness of fit.

### Genotypes as well as serum lipid levels

**Figure 1:**
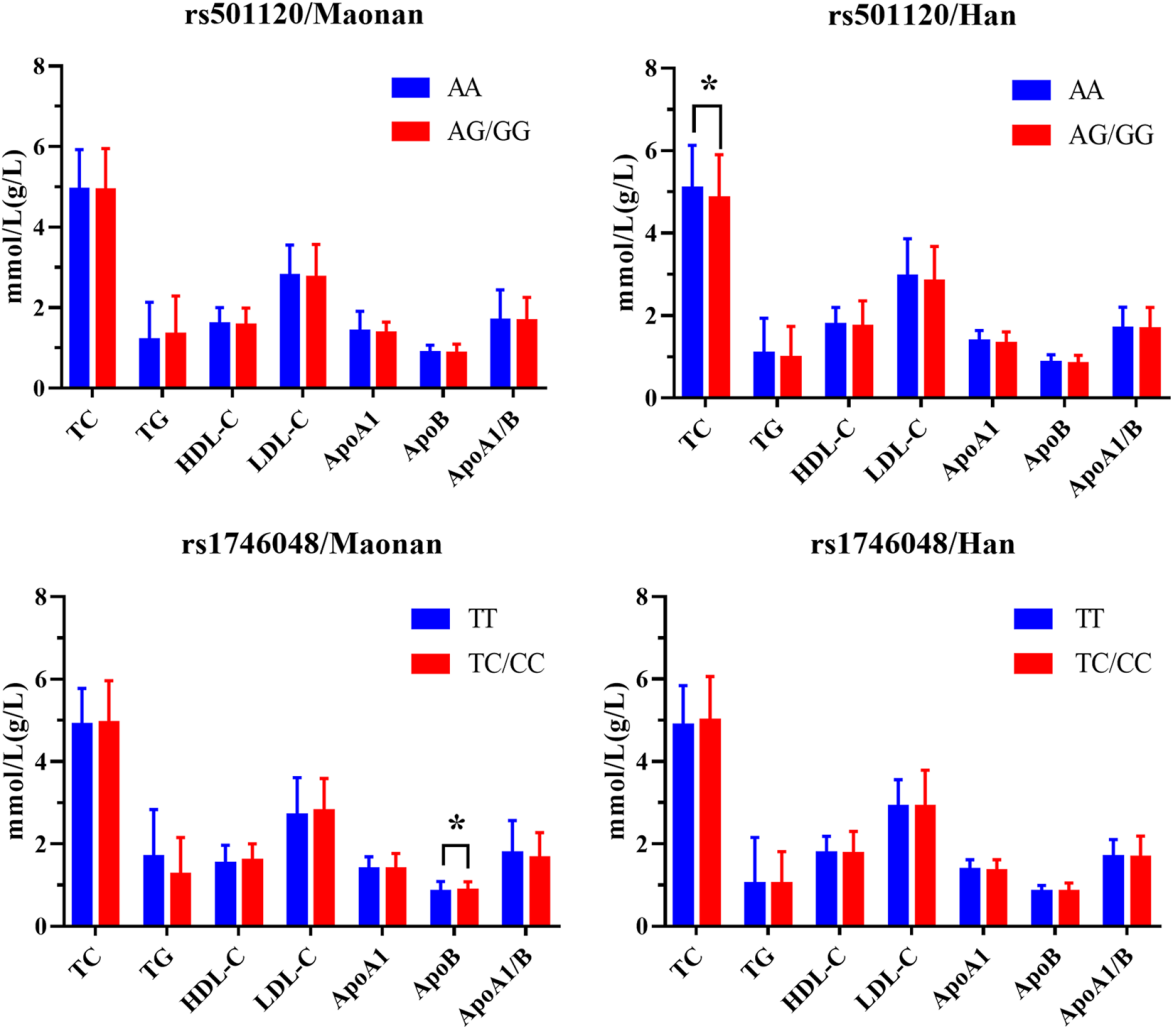
Comparison of the genotypes as well as serum lipid levels in Maonan and Han. *P < 0.025 signified statistical significance.

**Figure 2:**
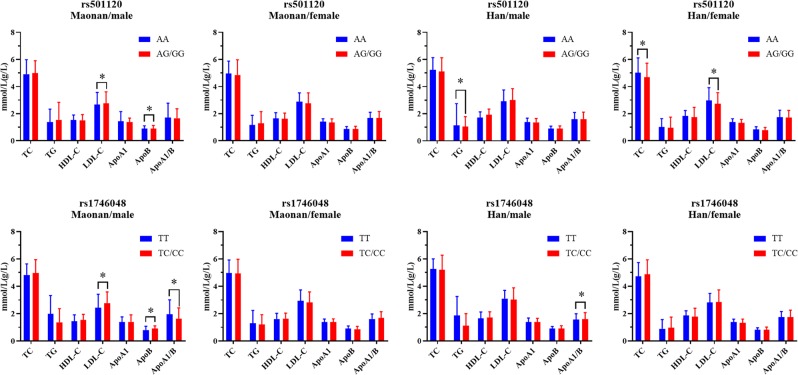
The *CXCL12* genotypes as well as serum lipid concentrations of males and females of both groups. *P < 0.025 signified statistical significance.

Figures 1 and 2 demonstrate the relationship among genotypes and serum lipid concentrations in both ethnic groups. The concentrations of TC in Han were dissimilar between the rs501120 genotypes (AA *vs*. AG/GG, *P* < 0.025 signified statistical significance after the Bonferroni correction). The concentrations of LDL-C plus ApoB in Maonan males were not similar amongst the rs501120 genotypes (*P* < 0.025 for each); the rs501120G allele carriers had greater LDL-C plus lesser ApoB concentrations compared to the rs501120G allele non-carriers. The concentrations of TG in Han males were dissimilar between rs501120 genotypes (*P* < 0.025); the rs501120G allele carriers had lesser TG concentrations compared to the rs501120G allele non-carriers. The concentrations of TC and LDL-C were dissimilar in Han females between the rs501120 genotypes (*P* < 0.025 for each); the rs501120G allele carriers had lesser TC plus LDL-C concentrations compared to the rs501120G allele non-carriers.

In addition, we also found that the concentrations of serum ApoB in Maonan were dissimilar between the rs1746048 genotypes (TT *vs*. TC/CC, *P* < 0.025) and the rs1746048C allele carriers had higher ApoB levels compared to the rs1746048C allele non-carriers. The concentrations of LDL-C and ApoB, and the ratio of ApoA1/ApoB were different in the Maonan males. Moreover, the rs1746048C allele carriers had greater LDL-C as well as ApoB levels, and lesser ApoA1/ApoB compared to the rs1746048C allele non-carriers. The ApoA1/ApoB ratio was different in Han males. The rs1746048C allele carriers had greater ApoA1/ApoB ratio compared to the rs1746048C allele non-carriers.

### Haplotypes and serum lipid levels

**Figure 3:**
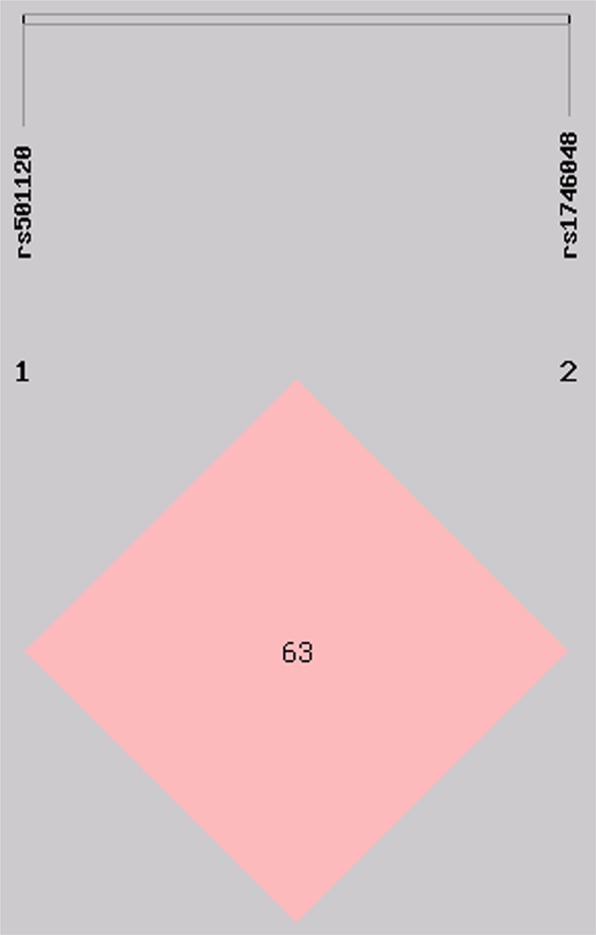
The linkage disequilibrium (LD) of the *CXCL12* rs501120 and rs1746048 SNPs in the combined population of Maonan and Han. The LD status was illustrated by the *r*^2^ = 0.63.

**Figure 4:**
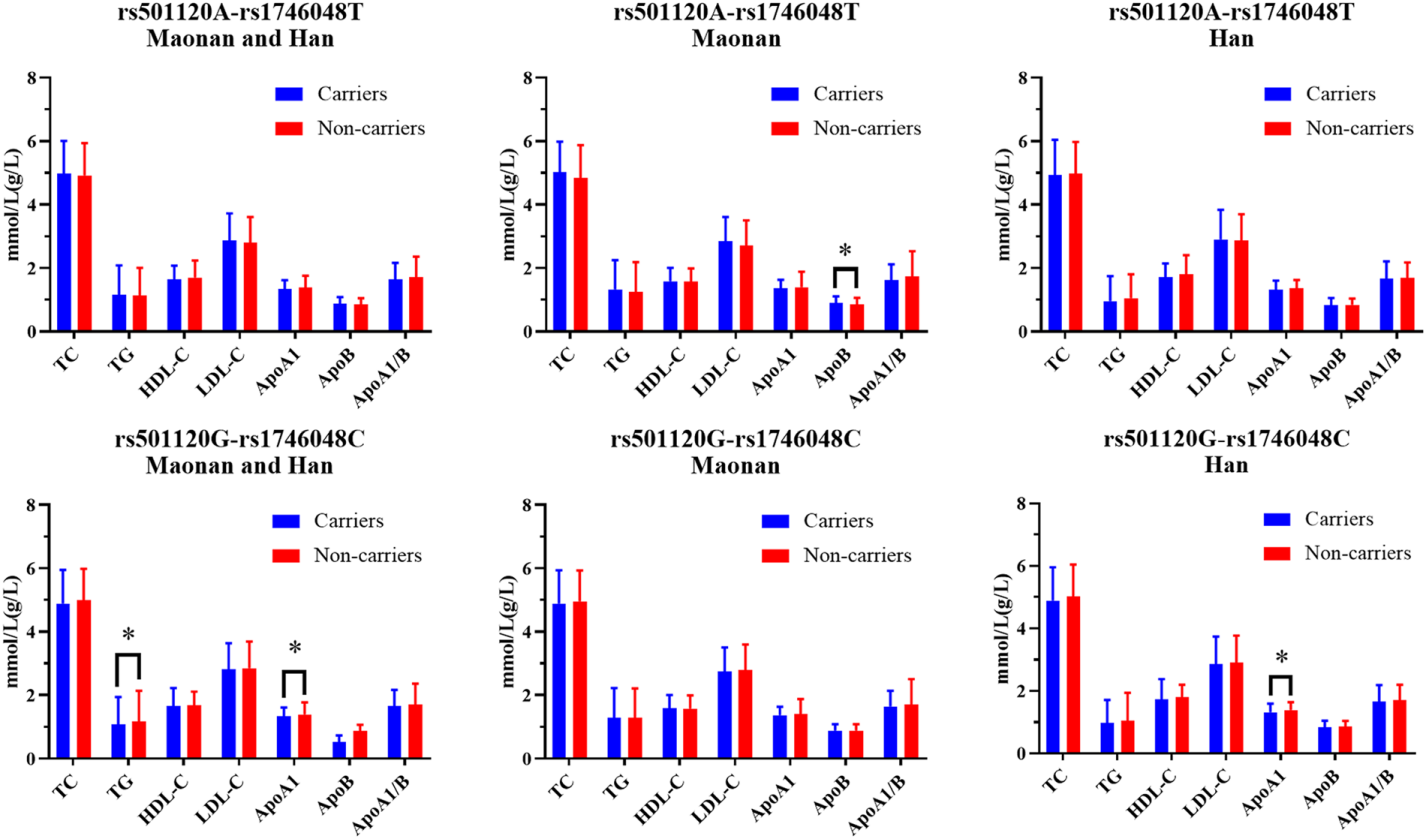
Serum lipid profiles of the *CXCL12* rs501120A-rs1746048T and rs501120G-rs1746048C haplotypes in both groups. *P < 0.025 signified statistical significance.

As presented in Figure 3, weak linkage disequilibrium (LD) was observed between the two SNPs (*r*^2^ = 0.63). Therefore, a haplotype analysis was conducted (Table 3). Four haplotypes were identified in the subjects of the current study. The haplotype of rs501120A-rs1746048C was the most common one (62.2%). The haplotype of rs501120A-rs1746048T was related with an augmented risk of hyperlipidemia (OR = 2.337, 95% CI = 1.252–4.361, *P* < 0.001), whereas the haplotype of rs501120G-rs1746048C was related with a diminished risk of hyperlipidemia (OR = 0.402, 95%CI = 0.234–0.690, *P* < 0.001). It also showed consistent association between the two haplotypes and serum TG, ApoA1 and ApoB concentrations. Multivariate logistic regression investigation indicated that the rs501120A-rs1746048T and rs501120G-rs1746048C haplotypes were associated with the occurrence of hyperlipidemia in Maonan and Han as per the stratified risk analyses including gender, BMI, alcohol consumption, diabetes as well as blood pressure (B*P*; *P*< 0.05; Table 4). The differences between the haplotypes and serum lipid profiles in both ethnic groups were as followed: the rs501120A-rs1746048T haplotype carriers had greater ApoB concentration than the rs501120A-rs1746048T haplotype non-carriers in the Maonan population. The rs501120G-rs1746048C haplotype carriers had lesser TG plus ApoA1 concentrations in Maonan and Han; as well as lesser ApoA1 concentrations in Han compared to the rs501120G-rs1746048C haplotype non-carriers (*P* < 0.025; Figure 4).

**Table 3:** *CXCL12* haplotype frequencies in the Maonan and Han populations [*n* (%)]

Haplotype	Total	Maonan	Han	P-value	OR (95%CI)
A-C	807 (62.3)	383 (62.4)	424(62.2)	0.931187	1.010 (0.807–1.265)
A-T	46 (3.5)	31 (5.0)	15 (2.2)	0.006183	2.337 (1.252–4.361)
G-C	69 (5.3)	19 (3.1)	50 (7.3)	0.000658	0.402 (0.234–0.690)
G-T	374 (28.9)	181 (29.5)	193(28.3)	0.632651	1.060 (0.834–1.349)

The order of haplotype composition was rs501120-rs1746048 SNPs.

**Table 4:** The *CXCL12* rs501120A-rs1746048T and rs501120G-rs1746048C haplotypes and hyperlipidemia in the Maonan and Han populations according to stratified risk factors.

Factor	Haplotype	OR(95%CI)_Maonan_	*P*_Maonan_	OR(95%CI)_Han_	*P*_Han_
**rs501120A-rs1746048T**					
**Gender**					
Male	Non-carriers	1	—	1	—
	Carriers	0.355(0.049–2.472)	0.723	1.010(0.097–3.827)	0.452
Female	Non-carriers	1	—	1	—
	Carriers	2.762(0.089–3.196)	0.729	5.338(0.261–6.230)	0.143
**Body mass index**					
<24 kg/m^2^	Non-carriers	1	—	1	—
	Carriers	0.491(0.108–3.182)	0.737	0.010(0.001–0.817)	0.354
≥24 kg/m^2^	Non-carriers	1	—	1	—
	Carriers	2.038(0.031–8.817)	0.044	3.683(1.232–7.665)	0.040
**Smoking**					
Non-smoker	Non-carriers	1	—	1	—
	Carriers	0.495(0.244–1.095)	0.083	1.813(0.064–5.127)	0.314
Smoker	Non-carriers	1	—	1	—
	Carriers	3.031(1.370–6.707)	< 0.001	1.055(0.001–1.558)	0.287
**Alcohol consumption**					
Non-alcohol consumer	Non-carriers	1	—	1	—
	Carriers	3.604(1.523–8.529)	0.311	3.046(0.307–4.167)	0.234
Alcohol consumer	Non-carriers	1	—	1	—
	Carriers	0.277(0.017–1.066)	0.003	0.033(0.003–0.015)	<0.001
**Diabetes**					
Non-diabetes	Non-carriers	1	—	1	—
	Carriers	2.164(0.064–9.891)	0.664	0.907(0.028–3.605)	0.216
Diabetes	Non-carriers	1	—	1	—
	Carriers	1.317(0.211–6.189)	0.958	0.101(0.002–0.438)	0.016
**Blood pressure**					
Normotensive	Non-carriers	1	—	1	—
	Carriers	4.604(0.858–9.972)	0.298	1.236(0.263–5.803)	0.554
Hypertension	Non-carriers	1	—	1	—
	Carriers	1.282(0.791–2.077)	0.313	0.737(0.455–1.194)	0.215
**rs501120G-rs1746048C**					
**Gender**					
Male	Non-carriers	1	—	1	—
	Carriers	0.336(0.001–8.170)	0.656	0.012(0.001–3.511)	0.133
Female	Non-carriers	1	—	1	—
	Carriers	3.702(0.012–8.924)	0.708	6.622(0.285–9.294)	0.134
**Body mass index**					
<24 kg/m^2^	Non-carriers	1	—	1	—
	Carriers	0.498(0.007–3.190)	0.743	0.061(0.006–0.745)	0.365
≥24 kg/m^2^	Non-carriers	1	—	1	—
	Carriers	2.006(0.031–8.459)	0.527	1.132(0.435–8.939)	0.034
**Smoking**					
Non-smoker	Non-carriers	1	—	1	—
	Carriers	0.589(0.209–1.669)	0.259	1.841(0.069–4.986)	0.307
Smoker	Non-carriers	1	—	1	—
	Carriers	1.696(0.767–3.746)	<0.001	0.044(0.001–1.148)	0.030
**Alcohol consumption**					
Non-alcohol consumer	Non-carriers	1	—	1	—
	Carriers	3.131(0.901–8.997)	0.989	2.131(1.492–8.315)	0.481
Alcohol consumer	Non-carriers	1	—	1	—
	Carriers	3.319(0.001–7.653)	0.988	0.046(0.014–0.106)	<0.001
**Diabetes**					
Non-diabetes	Non-carriers	1	—	1	—
	Carriers	0.466(0.015–4.750)	0.665	0.810(0.212–5.189)	0.180
Diabetes	Non-carriers	1	—	1	—
	Carriers	2.145(0.068–7.864)	0.713	0.012(0.001–0.482)	0.019
**Blood pressure**					
Normotensive	Non-carriers	1	—	1	—
	Carriers	4.164(0.791–9.273)	0.289	1.338(0.287–6.276)	0.598
Hypertension	Non-carriers	1	—	1	—
	Carriers	1.133(0.900–1.421)	0.003	0.793(0.491–1.281)	0.343

### Associated factors for serum lipid parameters

By performing the multivariable linear regression analysis on genotypes, environmental factors as well as serum lipid parameters in both groups, we found that the concentrations of ApoA1 were related with the rs501120 genotypes in both ethnic groups (*P* = 0.014). The concentrations of LDL-C, ApoB as well as the ratio of ApoA1 to ApoB were related with the rs1746048 genotype in Maonan males. The concentrations of TG in Han males (*P* = 0.038) and the concentrations of TC and ApoA1 in Han females were related with the rs1746048 genotypes (*P* = 0.049; *P* = 0.025, respectively; Table 5). As shown in Supplementary Table 1, serum lipid parameters were also interrelated with numerous environmental factors, for example gender, age, body weight, height, waist circumference, BMI, alcohol intake, cigarette smoking, BP, as well as blood glucose with ethnic variable among entire, Maonan and Han groups. Similarly, serum lipid parameters were also related with numerous environmental factors in entire population without ethnic variable (Supplementary Table 2).

**Table 5:** Correlation between the *CXCL12* rs501120 and rs1746048 genotypes and serum lipid levels in the Maonan and Han populations.

Lipid	Genotype	B	Std. error	*Beta*	*t*	*P*
**Maonan and Han**						
ApoA1	rs501120 genotype	−0.027	0.011	−0.090	−2.460	0.014
**Maonan/male**						
LDL-C	rs1746048 genotype	0.216	0.095	0.178	2.287	0.023
ApoB	rs1746048 genotype	0.061	0.023	0.200	2.584	0.011
ApoA1/ApoB	rs1746048 genotype	−0.191	0.092	−0.163	−2.084	0.039
**Han/male**						
TG	rs501120 genotype	−0.288	0.138	−0.181	−2.095	0.038
**Han/female**						
TC	rs501120 genotype	−0.131	0.066	−0.129	−1.978	0.049
ApoA1	rs501120 genotype	−0.036	0.016	−0.147	−2.249	0.025

TC, total cholesterol; HDL-C, high-density lipoprotein cholesterol; LDL-C, low-density lipoprotein cholesterol; ApoA1, apolipoprotein A1; ApoB, apolipoprotein B; ApoA1/ApoB, the ratio of apolipoprotein A1 to apolipoprotein B; B and Std. error, unstandardized coefficients; Beta, standardized coefficient.

## Discussion

This study indicated that the effects of the two *CXCL12* SNPs on serum lipid profiles were dissimilar: (a) the concentrations of TC in the Han were dissimilar between the rs501120 genotypes, and the ApoB concentrations in the Maonan were dissimilar between the rs1746048 genotypes; (b) the concentrations of LDL-C as well as ApoB in Maonan males, TG level in Han males, TC and LDL-C concentrations in Han females differed between the rs5011120 genotypes; and (c) the concentrations of LDL-C plus ApoB along with ApoA1/ApoB ratio in the Maonan males and ApoA1/ApoB ratio in the Han males differed between the rs1746048 genotypes. Both rs501120 and rs1746048 SNPs were associated with serum LDL-C along with ApoB concentrations.

On the other hand, we established that the rs501120G allele carriers had greater LDL-C as well as lesser ApoB concentrations than the rs501120G allele non-carriers in Maonan males but not in Han males, while the G allele carriers had lesser LDL-C levels than the G allele non-carriers in Han females. There was not any substantial change between the concentrations of HDL-C plus ApoA1 and the genotypes of the two SNPs in both groups. It is well-acknowledged that hyperlipidemia is a complicated syndrome influenced by environmental as well as genetic factors and their interactions^9^. Preceding reports about family and twins have revealed that in large populations around 50% variation of the serum lipid profiles is genetically measured^30–34^. Serum LDL-C concentration is related with the advancement of CAD and can cause atherosclerotic cardiovascular disease (ASCVD)^35^. Carr *et al*. showed that ApoB was the primary organizing protein component of some serum lipids and was required for the formation of these lipids and lipoproteins, and it could be recognized as a measure of the number of atherogenic lipoproteins^36^. In other words, both LDL-C and ApoB may have a relationship with CAD or ASCVD, which is consistent with the connection amongst the SNPs and serum lipid profiles in the current study.

In addition, the results of the genotypic frequencies about the two SNPs in the both Chinese populations were not analogous to those demonstrated in other populations acquired from the International 1000 Genomes database. The allelic frequencies of the two SNPs in different populations are shown in Supplementary Table 3. This study showed substantial changes in the genotypic incidences of the rs501120 and rs1746048 SNPs between both ethnic groups. The minor allele frequency (MAF) of the rs501120 and rs1746048 SNPs was lesser in the Maonan compared to the Han. According to these outcomes, we could infer that the incidence of the *CXCL12* rs501120 and rs1746048 SNPs may display racial/ethnic dissimilarity. Therefore, we strongly believe that the dissimilarities in genotypic frequencies between both ethnic groups may occur due to their different genetic background as well as environmental exposure.

Diet, lifestyle, obesity, physical activity, hypertension as well as the environmental factors would have an essential involvement in changing serum lipid levels^37–40^. The Maonan and Han populations have different dietary habits. Rice is the Maonan people’s primary food. They also like strong flavor. They adore eating spicy and acid or sour foods that contain lots of oil and salt. It is likely that the preference for this high carbohydrate diet contributes to Maonan people having a higher weight and waist circumference than in Han people. In the meanwhile, foods high in fat and salt can cause hypertension, higher levels of serum TG as well as ApoB in the Maonan compared to the Han. Many previous studies have confirmed that diet, by itself, is obligated to the inconsistency on serum lipid levels^41,42^. In the current study, some environmental factors, for example age, waist circumference, BMI, alcohol intake, cigarette smoking, BP, and blood glucose showed correlations with serum lipid parameters. It is widely believed that high-fat diets, particularly comprising great amounts of saturated fatty acids, produce elevated serum cholesterol levels and may make people susceptible to CAD^43^. In the present study, however, we didn’t find difference in the percentage of alcohol consumption between both ethnic groups, but we found some relationships between serum lipid parameters and alcohol consumption in both ethnic groups. Moreover, alcohol consumption, an important factor that influences serum lipid levels has been a hot topic and popular focus in recent years^44^. Onat *et al*. also conducted a research about alcohol and serum lipids. It showed that increased TG, LDL-C as well as ApoB levels existed in the males of their subjects with alcohol, and alcohol consumption in females reduced TG but did not alter LDL-C or ApoB levels^45^. When the ethnic variable was excluded from the predictors in the multiple linear regression evaluation, we found that the data such as the standard coefficient had some changes. As for the mechanism of mutation causing serum lipid changes, it is not clear in our study. But the environmental factor has a great influence on blood lipid levels, and can even change the genetic effects of genes. For the *CXCL12* and its expression, a previous study found that the *CXCL12* was less hypomethylated in patients with CAD, and the *CXCL12* methylation was negatively correlated with its expression^46^. Therefore, the further modification of the association between genetic variation and serum lipid concentrations may attribute to the exposure to diverse lifestyle as well as environmental factors.

Vital intra- and inter-genetic linkage disequilibrium (LD) relations were also noted in the current study. The LD pattern of the two detected *CXCL12* SNPs was weak and low in our study populations, and it specified the functional dependences of the encoded relevant proteins, even though the two SNPs were located in non-coding region. The relationship amongst the *CXCL12* polymorphisms and serum lipid levels in both ethnic groups could be partly explained by haplotype analysis. The haplotype of rs501120A-1746048C was the most common and signified around 62.3% of the samples. The rs501120A-rs1746048T haplotype increased and the rs501120G-rs1746048C haplotype decreased the risk of hyperlipidemia. Moreover, carriers of rs501120A-rs1746048T haplotype had increased serum ApoB concentrations, whereas carriers of rs501120G-rs1746048C haplotype had decreased serum TG and ApoA1 concentrations. We should make a statement that we only described the rs501120 G allele in the specific research populations (the Maonan male and Han female) with the allele decreasing LDL-C levels (decreasing risk factors), not for all mankind; it might be the gender difference that led to these results. Whether other alleles could increase or decrease serum lipid levels, the current research results had not yet been strongly supported. The description in the haplotype analysis was only discussed from the results of statistical analysis. If more explanations are needed, such as biological aspects, more in-depth experimental support is needed. Although there was only a tendency to risk allele of the rs501120 SNP in European patients with Moyamoya disease^47^, we established that haplotypes of the two *CXCL12* SNPs might partly elucidate considerable serum lipid variation compared to single SNP alone in our study populations.

Our analysis benefited from a strong hypothesis and the indicators from other researchers of our group, a good sample size and population for study, and compared with clinical samples, there was less sample related issue. Nevertheless, the shortcomings and limitations are: (1) the amount of the study samples is relatively small; (2) some general characteristics could influence the concentrations of serum lipids despite adjustment in the data analysis. (3) information about other SNPs near *CXCL12* may be missing; (4) the selection of the SNPs was limited; (5) our study was originally an observational finding, and it would be impossible to recollect blood samples for institutional research because of national protection policies.

Through the collection of samples, blood biochemical testing, genotyping and gene sequencing, and statistical analysis, we concluded that the genotypic as well as allelic incidences of the *CXCL12* rs501120 and rs1746048 SNPs were not similar between Maonan and Han. There were ethnic- as well as sex-specific associations among the *CXCL12* SNPs and serum lipid levels. We detected four haplotypes and the commonest one was the rs501120A-rs1746048C haplotype. Two haplotypes of rs501120A-rs1746048T and rs501120G-rs1746048C showed an association with serum lipid traits. All these differences may partly attribute to the *CXCL12* SNPs, their haplotypes, environmental factors as well as gene-environment interactions.

## Materials and Methods

### Participants

We enrolled 750 unrelated individuals (324 males, 43.2% and 426 females, 56.8%; 25–80 year; mean age 55.71 ± 15.10 years) of Maonan and 744 unrelated individuals (270 males, 36.29% and 474 females, 63.71%; 25–80 years; mean age 54.08 ± 15.49 years) of Han. They were arbitrarily elected from our earlier stratified randomized samples. They were farming personnel from Huanjiang Maonan Autonomous County, China. All subjects were basically in good health with no history of any serious diseases. They were not taking any medicines that could influence serum lipid concentrations. All experimental techniques were accomplished as per the appropriate ethical standards. Protocols were permitted by the Ethics Committee of the First University Hospital of Guangxi Medical University (No: Lunshen-2014-KY-Guoji-001; March 7, 2014). All subjects signed the written informed consent.

### Epidemiological survey

Epidemiological questionnaires were developed according to the International Standardized Survey of Cardiovascular Diseases^48^. The clinical data were collected by the specialists (relevant doctors, postgraduate students and nurses) trained from the First Affiliated Hospital, Guangxi Medical University. Gender, age, medical history, smoking and drinking history, family history, history of medications related to CVD, occupation, education, workforce, eating habits, etc. of the participants were recorded in detail. According to the amount, degree and type of alcohol, the intake of alcohol consumption was collected in the past 12 months. Alcohol intake was classified into groups of grams of alcohol per day: 0 (non-drinker), ≤25 and >25^49^. Similarly, cigarettes smoking was also classified into three groups: 0 cigarette per day (non-smoker), ≤20 cigarettes per day and >20 cigarettes per day^50^. Other parameters such as height, weight, BMI, BP as well as waist circumference were also determined sequentially. The methods for measuring relevant parameters referred to previous research methods^51^.

### Biochemical measurements

Blood sample collection: All subjects fasted for more than 12 hours the day before blood collection. The subjects took the sitting position and evacuated the fasting venous blood in the morning. A well-trained professional nurse took the blood sample of 5 ml from the elbow vein, and 2 ml was placed in a non-anticoagulated tube to solidify and separate the serum for blood biochemical measurements. Another 3 ml was treated with a special anticoagulant (ACD) containing 13.20 g/L trisodium citrate, 14.70 g/L glucose and 4.80 g/L citric acid for further removal of the DNA and it was stored in a refrigerator at −20 °C for further experiment after testing its concentration and purity. Blood biochemical indices detection: The Hitachi 7170 automatic biochemical analyzer was utilized to measure the serum biochemical indices of the collected blood samples: (1) Serum TC and TG levels were determined using enzyme labeling; (2) Serum ApoA1 as well as ApoB levels were determined using immunoturbidimetry; and (3) Serum HDL-C as well as LDL-C levels were determined by an enzyme-linked immunoassay. The outcomes were verified in the Clinical Science Experiment Center of the First Affiliated Hospital, Guangxi Medical University^40,52–55^. In addition, fasting blood glucose from fingertip peripheral blood was also determined by a Roche blood glucose meter^54,55^.

### DNA amplification as well as genotyping

(1) Genomic DNA was acquired from peripheral blood leukocytes of the samples as per the phenol-chloroform method^52,53^. (2) Extracted DNA was kept at −20 °C. (3) Genotyping of the *CXCL12* rs501120 and rs1746048 SNPs was accomplished by PCR-RFLP. The specific designs of PCR amplification systems are shown in Supplementary Tables 4 and 5. (4) PCR was implemented as follows: 95 °C for 5 min, followed by 30 s denaturing at 95 °C, 30 s of annealing at 59 °C for the rs501120 SNP or at 57 °C for the rs1746048 SNP, and 40 s of elongation at 72 °C for 33 cycles. The last step ended at 72 °C for 7 min of a concluding extension. (5) Following the electrophoresis on a 2.0% agarose gel containing 0.5 μg/mL ethidium bromide (EtBr), the PCR products were observed under ultraviolet light. (6) The digestions were conducted at 37 °C for 75 min for the rs501120 SNP and at 60 °C for 30 min for the rs1746048 SNP. The specific compositions of restriction enzyme digestion are shown in Supplementary Table 6. (7) After the amplified DNA was digested with restriction enzymes, the genotypes were recognized by agarose gel electrophoresis with 2% EtBr and detected using UV light. (8) Researchers who did not know the results of epidemiology and serum lipid profiles recorded the outcomes of genotyping. Twelve samples (each genotype for two, namely 6 samples for the Maonan and 6 samples for the Han) distinguished by the PCR-RFLP underwent direct sequencing examination by means of ABI Prism 3100 (Applied Biosystems).

### Diagnostic criteria

Following values were considered as the normal values: serum TC: 3.10–5.17 mmol/L, TG: 0.56–1.70 mmol/L, HDL-C: 1.16–1.42 mmol/L, LDL-C: 2.70–3.10 mmol/L, ApoA1: 1.20–1.60 g/L, ApoB: 0.80–1.05 g/L, and the ApoA1/ApoB ratio: 1.00–2.50. When the participant’s serum TC concentration was more than 5.17 mmol/L and/or his or her serum TG level was beyond 1.70 mmol/L, he or she was diagnosed as hyperlipidemic^56^. Hypertension was diagnosed by the following criteria: SBP ≥ 140 mmHg and/or DBP ≥ 90 mmHg were determined twice in the absence of antihypertensive drugs and averaged after three consecutive measurements^57^. The value of BMI in normal weight, overweight and obesity ranged from <24, 24–28 and >28 kg/m^2^, respectively^58^. According to the WHO diagnostic criteria for diabetes, type 2 diabetes was diagnosed by the following criteria: (1) fasting glucose ≥7.0 mmol/L; (2) 2 hours postprandial glucose ≥11.1 mmol/L; or (3) self-reported opinion of diabetes or usage of anti-diabetic medicines^59^.

### Statistical analyses

The analysis was done using SPSS 21.0 (USA). The quantitative variables of normal distribution were mentioned as mean ± standard deviation, for example age, height, weight, BMI, waist circumference, BP, glucose, the concentrations of serum TC, HDL-C, LDL-C, ApoA1, ApoB and the ratio of ApoA1/ApoB; whereas serum TG concentrations were mentioned as medians plus interquartile ranges for its skewed distribution. Direct calculation of the allele frequency and the genotype distribution among groups was assessed by the chi-square test. The Student’s unpaired *t*-test was utilized for the comparison of general characteristics between the two ethnic groups. The relationship among genotypes and serum lipid parameters was evaluated by covariance analysis (ANCOVA). When each SNP related with serum lipid profiles fitted the value of *P* < 0.025 (matching *P* < 0.05 after adjusting for two independent assessments by the Bonferroni correction), it reflected statistical significance. The method to evaluate the haplotype frequencies as well as pair-wise LD amongst the identified SNPs was Haploview (USA, version 4.2). The range of the *r*^2^ values was 0.30–0.80 as significant LD. Haplotype identification and the association of haplotype and the risk of hyperlipidemia were analyzed in the entire study population, and the stratified risk analysis was conducted from the separated population of Maonan and Han. To evaluate the connection between the risk of hyperlipidemia and genotypes, we considered performing an unconditional logistic regression analysis. To investigate the association among the genotypes and numerous environmental factors with serum lipid levels, we conducted a multivariable linear regression examination with stepwise modeling (with or without ethnic variable), defining the common homozygote genotype as 1, heterozygote genotype as 2 and rare homozygote genotype as 3. *P* value < 0.05 signified statistical significance.

## Supplementary information

Supplementary information 
