# Supplementary material for: The CXCL12 SNPs and their haplotypes are associated with serum lipid traits
Source: Sci Rep. 2019 Dec 20;9:19524. doi: 10.1038/s41598-019-55725-3 (PMC6925251; doi:10.1038/s41598-019-55725-3)
Supplement: Supplementary file 1 — Supplementary information [file 41598_2019_55725_MOESM1_ESM.docx]

**The *CXCL12* SNPs and their haplotypes are associated with serum lipid traits**

Ling Qiu^1^, Rui-Xing Yin^1,2,3^, Rong-Jun Nie^1^, Xi-Jiang Hu^1^, Eksavang Khounphinith^1^ & Fen-Han Zhang^1^

^1^ Department of Cardiology, Institute of Cardiovascular Diseases, the First Affiliated Hospital, Guangxi Medical University, Nanning 530021, Guangxi, People’s Republic of China

^2^ Guangxi Key Laboratory Base of Precision Medicine in Cardio-cerebrovascular Disease Control and Prevention, Nanning 530021, Guangxi, People’s Republic of China

^3^ Guangxi Clinical Research Center for Cardio-cerebrovascular Diseases, Nanning 530021, Guangxi, People’s Republic of China

Correspondence and requests for materials should be addressed to R.-X.Y. (email: yinruixing@163.com)

*CXCL12* SNPs and serum lipid traits

Ling Qiu, danielhill@foxmail.com

Rui-Xing Yin, yinruixing@163.com

Rong-Jun Nie, nrj2001@163.com

Xi-Jiang Hu, huxijianght@126.com

Eksavang Khounphinith, Ek_cupid@hotmail.com

Fen-Han Zhang, zfenhan@163.com


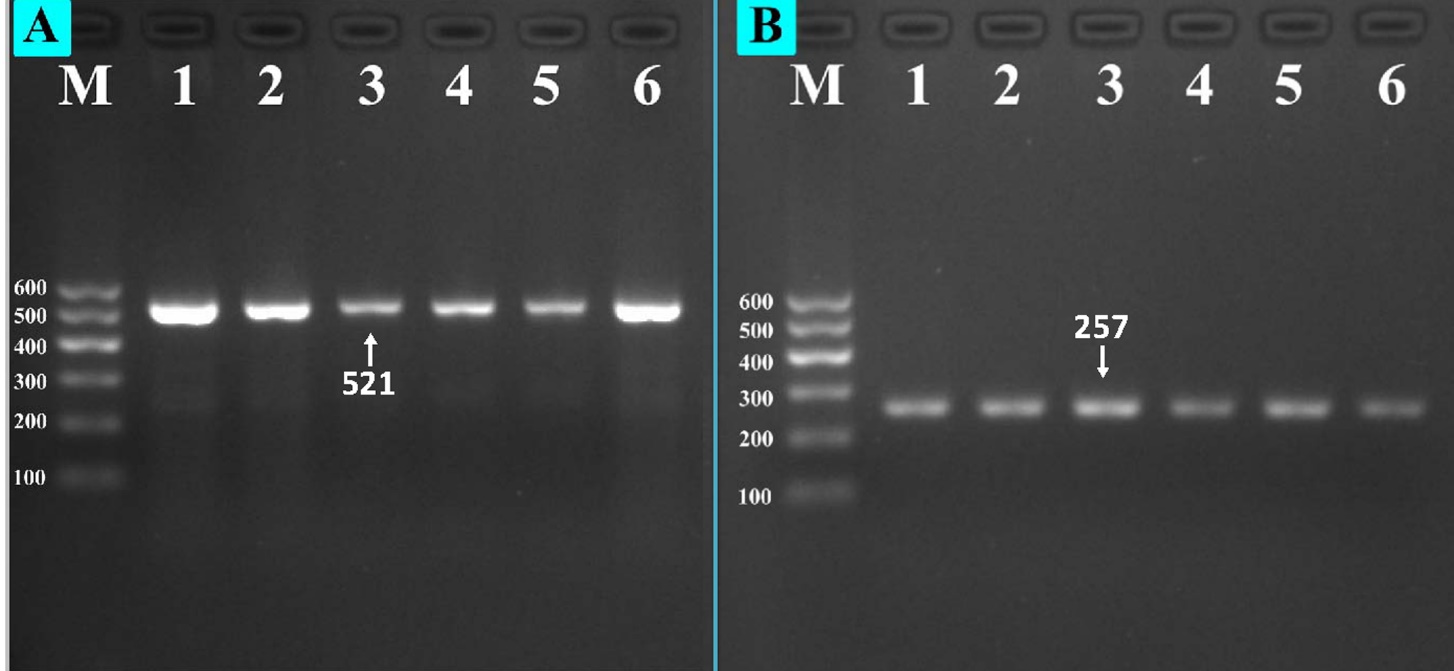


**Supplementary Figure 1.** Electrophoresis of polymerase chain reaction products of the samples. Lane M was the 100 bp marker ladder; **(A) *CXCL12* rs501120 SNP:** lanes 1-6 were samples, the 521 bp bands were the target genes; **(B) *CXCL12* rs1746048 SNP:** lanes 1-6 were samples, the 257 bp bands were the target genes.


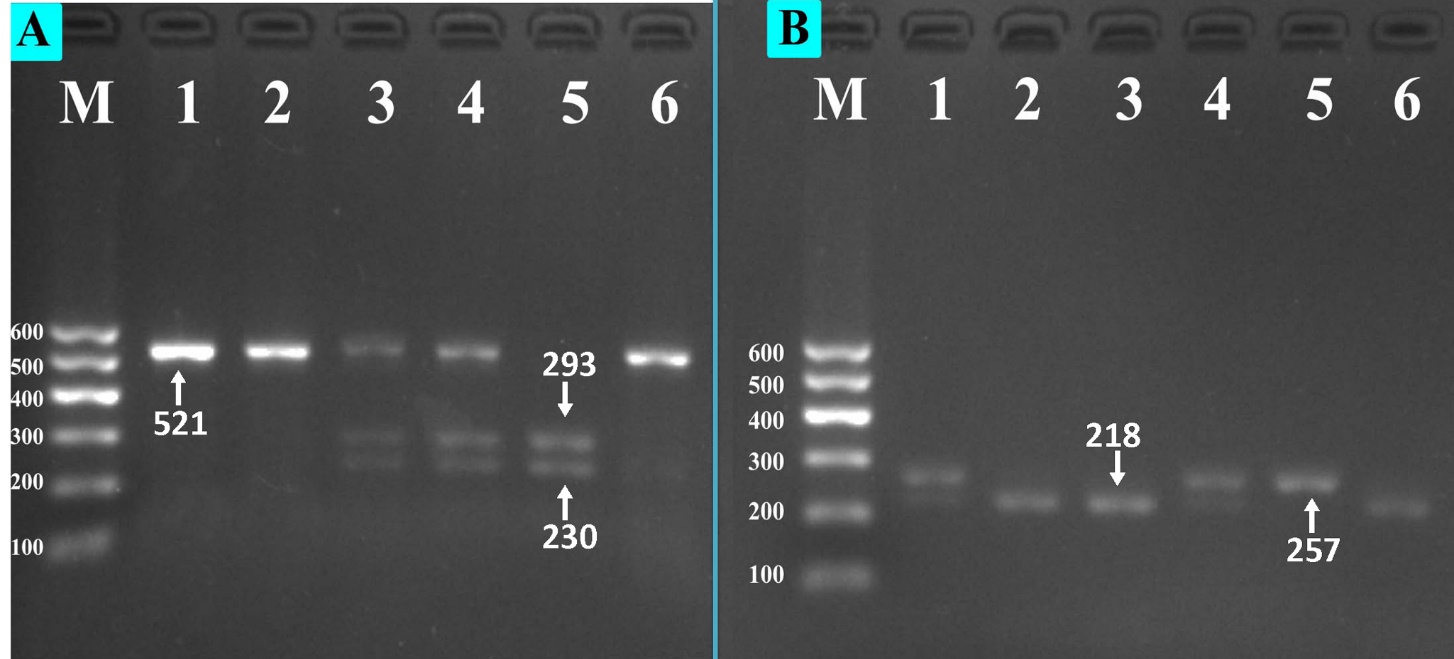


**Supplementary Figure 2.** Genotyping of the *CXCL12* rs501120 and rs1746048 SNPs. **(A) *CXCL12* rs501120 SNP:** Lane M, 100 bp marker ladder; lanes 1, 2 and 6, AA genotype (521 bp); lanes 3 and 4, AG genotype (521-, 293- and 230-bp); and lane 5, GG genotype (293- and 230-bp). **(B) *CXCL12* rs1746048 SNP:** Lane M, 100 bp marker ladder; lane 5, TT genotype (257 bp); lanes 1 and 4, TC genotype (257-, 218- and 39-bp); and lanes 2, 3 and 6, CC genotype (218- and 39-bp). The 39-bp fragments were invisible in the gel owing to their fast migration speed.


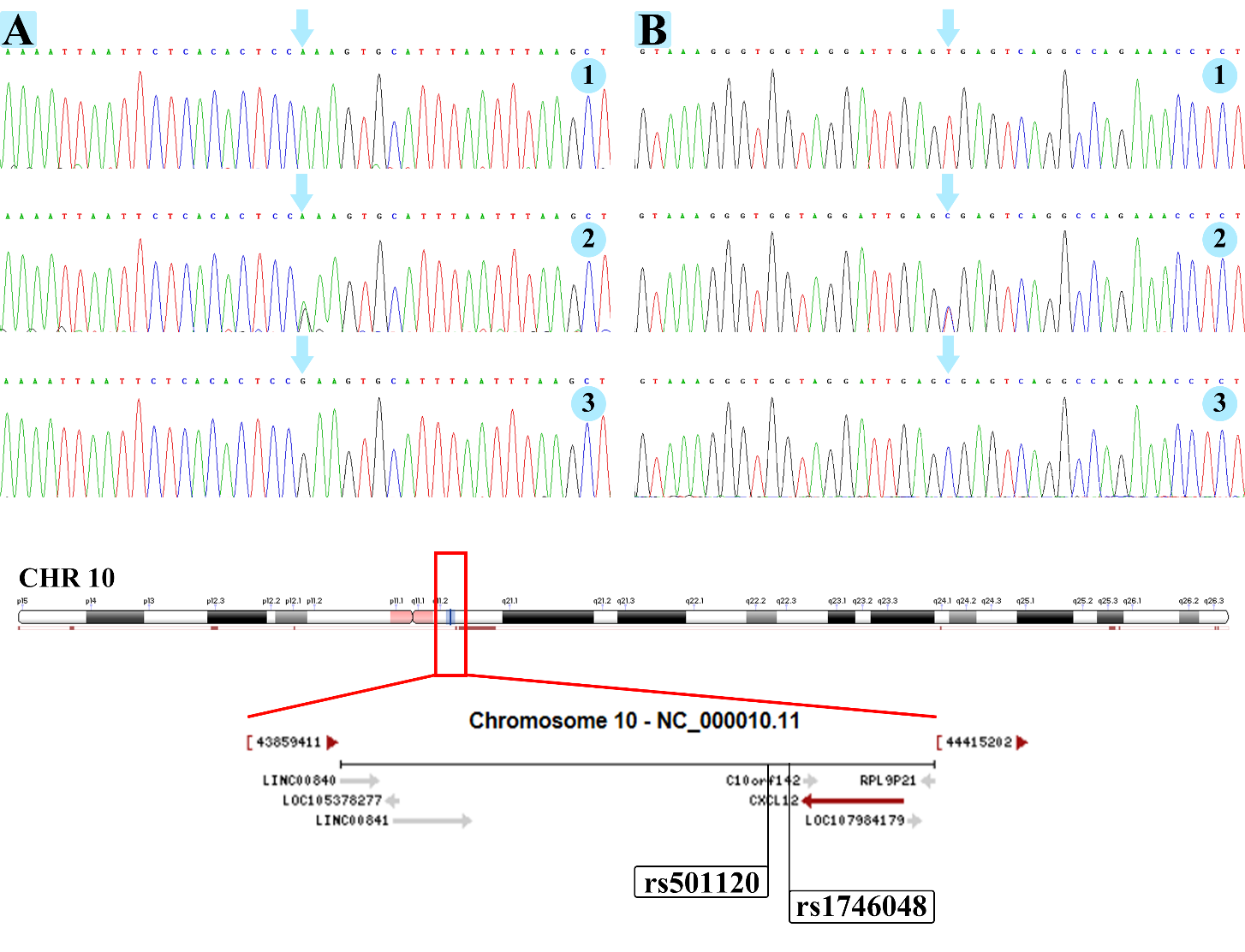


**Supplementary Figure 3**. A part of the nucleotide sequences of the *CXCL12* rs501120 and rs1746048 SNPs and the location of the *CXCL12* on Chromosome 10. **(A) *CXCL12* rs501120 SNP:** 1, AA genotype; 2, AG genotype; and 3, GG genotype. **(B) *CXCL12* rs1746048 SNP:** 1, TT genotype; 2, TC genotype; and 3, CC genotype.

**Supplementary Table 1** Relationship between serum lipid parameters and relative factors in the Maonan and Han populations

| Lipid | Risk factor | B | Std. error | *Beta* | *t* | *P* |
| --- | --- | --- | --- | --- | --- | --- |
| Maonan and Han | |  |  |  |  |  |
| TC | WC | 0.016 | 0.004 | 0.140 | 3.900 | 1.004E-04 |
|  | Age | 0.012 | 0.002 | 0.180 | 6.937 | 5.973E-12 |
|  | BMI | 0.031 | 0.011 | 0.098 | 2.752 | 0.006 |
|  | Ethnic group | 0.135 | 0.052 | 0.066 | 2.608 | 0.009 |
|  | DBP | 0.005 | 0.002 | 0.059 | 2.226 | 0.026 |
| TG | WC | 0.044 | 0.007 | 0.229 | 6.056 | 1.771E-09 |
|  | AC | 0.005 | 0.001 | 0.107 | 4.001 | 6.615E-05 |
|  | CS | 0.016 | 0.005 | 0.087 | 3.193 | 0.001 |
|  | Glucose | 0.099 | 0.028 | 0.085 | 3.489 | 4.992E-04 |
|  | Weight | 0.017 | 0.007 | 0.101 | 2.616 | 0.009 |
| HDL-C | WC | -0.012 | 0.001 | -0.220 | -8.484 | 5.224E-17 |
|  | Ethnic group | 0.138 | 0.024 | 0.143 | 5.694 | 1.499E-08 |
|  | AC | 0.002 | 3.952E-04 | 0.163 | 5.910 | 4.235E-09 |
|  | Gender | 0.088 | 0.028 | 0.089 | 3.119 | 0.002 |
|  | Pulse pressure | -0.002 | 7.729E-04 | -0.086 | -3.058 | 0.002 |
|  | Age | 0.003 | 0.001 | 0.091 | 3.273 | 0.001 |
| LDL-C | Age | 0.012 | 0.001 | 0.224 | 8.876 | 1.964E-18 |
|  | BMI | 0.028 | 0.009 | 0.109 | 3.078 | 0.002 |
|  | Ethnic group | 0.170 | 0.042 | 0.102 | 4.002 | 6.588E-05 |
|  | AC | -0.002 | 0.001 | -0.094 | -3.730 | 1.987E-04 |
|  | WC | 0.009 | 0.003 | 0.095 | 2.645 | 0.008 |
| ApoA1 | AC | 0.002 | 2.675E-04 | 0.172 | 6.134 | 1.095E-09 |
|  | WC | -0.006 | 0.001 | -0.161 | -6.183 | 8.125E-10 |
|  | Ethnic group | -0.050 | 0.016 | -0.078 | -3.049 | 0.002 |
|  | CS | 0.003 | 0.001 | 0.085 | 3.018 | 0.003 |
| ApoB | WC | 0.006 | 0.001 | 0.240 | 7.060 | 2.555E-12 |
|  | Age | 0.002 | 3.342E-04 | 0.155 | 6.105 | 1.311E-09 |
|  | BMI | 0.008 | 0.002 | 0.123 | 3.653 | 2.685E-04 |
|  | DBP | 0.001 | 3.946E-04 | 0.064 | 2.528 | 0.012 |
|  | Glucose | 0.008 | 0.003 | 0.055 | 2.260 | 0.024 |
| ApoA1/ApoB | WC | -0.014 | 0.002 | -0.217 | -6.058 | 1.743E-09 |
|  | AC | 0.002 | 4.805E-04 | 0.134 | 4.764 | 2.085E-06 |
|  | Age | -0.004 | 0.001 | -0.112 | -4.460 | 8.808E-06 |
|  | BMI | -0.019 | 0.006 | -0.106 | -3.066 | 0.002 |
|  | Glucose | -0.023 | 0.010 | -0.057 | -2.288 | 0.022 |
|  | CS | 0.006 | 0.002 | 0.090 | 3.014 | 0.003 |
|  | Gender | 0.092 | 0.036 | 0.079 | 2.559 | 0.011 |
| Maonan |  |  |  |  |  |  |
| TC | Age | 0.017 | 0.003 | 0.254 | 6.191 | 9.907E-10 |
|  | Weight | 0.032 | 0.004 | 0.337 | 8.292 | 5.207E-16 |
|  | Gender | 0.308 | 0.081 | 0.151 | 3.792 | 1.613E-04 |
|  | Pulse pressure | -0.004 | 0.002 | -0.082 | -2.024 | 0.043 |
| TG | Weight | 0.037 | 0.009 | 0.235 | 4.144 | 3.813E-05 |
|  | AC | 0.008 | 0.002 | 0.164 | 4.138 | 3.898E-05 |
|  | WC | 0.026 | 0.009 | 0.144 | 2.722 | 0.007 |
|  | Glucose | 0.094 | 0.044 | 0.072 | 2.148 | 0.032 |
|  | CS | 0.018 | 0.007 | 0.103 | 2.492 | 0.013 |
|  | Gender | 0.301 | 0.146 | 0.090 | 2.066 | 0.039 |
| HDL-C | WC | -0.005 | 0.002 | -0.114 | -2.103 | 0.036 |
|  | AC | 0.002 | 4.321E-04 | 0.197 | 5.180 | 2.862E-07 |
|  | Pulse pressure | -0.006 | 0.001 | -0.262 | -4.052 | 5.610E-05 |
|  | Gender | 0.135 | 0.033 | 0.162 | 4.077 | 5.062E-05 |
|  | BMI | -0.026 | 0.007 | -0.200 | -3.875 | 1.160E-04 |
|  | SBP | 0.003 | 0.001 | 0.170 | 2.608 | 0.009 |
|  | Glucose | -0.028 | 0.011 | -0.088 | -2.563 | 0.011 |
| LDL-C | Age | 0.016 | 0.002 | 0.297 | 7.262 | 9.683E-13 |
|  | AC | -0.004 | 0.001 | -0.158 | -4.095 | 4.695E-05 |
|  | Weight | 0.019 | 0.003 | 0.252 | 6.227 | 7.963E-10 |
|  | Gender | 0.182 | 0.071 | 0.111 | 2.566 | 0.010 |
|  | Pulse pressure | -0.004 | 0.002 | -0.085 | -2.122 | 0.034 |
| ApoA1 | WC | -0.006 | 0.001 | -0.145 | -3.963 | 8.128E-05 |
|  | AC | 0.002 | 3.665E-04 | 0.161 | 4.465 | 9.236E-06 |
|  | Pulse pressure | -0.002 | 0.001 | -0.099 | -2.747 | 0.006 |
|  | Glucose | -0.027 | 0.010 | -0.093 | -2.570 | 0.010 |
| ApoB | WC | 0.004 | 0.001 | 0.182 | 3.268 | 0.001 |
|  | Age | 0.003 | 4.884E-04 | 0.252 | 7.001 | 5.700E-12 |
|  | Weight | 0.005 | 0.001 | 0.248 | 4.039 | 5.915E-05 |
|  | Gender | 0.054 | 0.016 | 0.130 | 3.383 | 7.555E-04 |
| ApoA1/ApoB | WC | -0.021 | 0.002 | -0.314 | -9.073 | 1.019E-18 |
|  | AC | 0.003 | 0.001 | 0.183 | 5.287 | 1.637E-07 |
|  | Age | -0.007 | 0.001 | -0.164 | -4.815 | 1.781E-06 |
| Han |  |  |  |  |  |  |
| TC | Age | 0.011 | 0.003 | 0.160 | 4.266 | 2.255E-05 |
|  | WC | 0.023 | 0.005 | 0.168 | 4.583 | 5.399E-06 |
|  | AC | 0.003 | 0.001 | 0.097 | 2.506 | 0.012 |
|  | DBP | 0.009 | 0.003 | 0.111 | 2.995 | 0.003 |
|  | Height | -0.020 | 0.006 | -0.146 | -3.411 | 6.834E-04 |
|  | Gender | -0.282 | 0.097 | -0.131 | -2.911 | 0.004 |
| TG | WC | 0.062 | 0.008 | 0.286 | 7.921 | 8.749E-15 |
|  | CS | 0.023 | 0.007 | 0.116 | 3.353 | 8.396E-04 |
|  | Glucose | 0.096 | 0.037 | 0.091 | 2.613 | 0.009 |
|  | DBP | 0.011 | 0.005 | 0.082 | 2.293 | 0.022 |
| HDL-C | Weight | -0.012 | 0.002 | -0.203 | -5.446 | 7.053E-08 |
|  | AC | 0.002 | 0.001 | 0.120 | 3.205 | 0.001 |
| LDL-C | Age | 0.012 | 0.002 | 0.217 | 5.973 | 3.637E-09 |
|  | BMI | 0.045 | 0.009 | 0.178 | 5.071 | 5.025E-07 |
|  | Glucose | 0.052 | 0.020 | 0.095 | 2.616 | 0.009 |
| ApoA1 | AC | 0.002 | 3.218E-04 | 0.274 | 7.302 | 7.405E-13 |
|  | Weight | -0.006 | 0.001 | -0.209 | -5.777 | 1.122E-08 |
|  | CS | 0.004 | 0.001 | 0.140 | 3.768 | 1.781E-04 |
| ApoB | WC | 0.008 | 0.001 | 0.307 | 8.722 | 1.847E-17 |
|  | SBP | 0.002 | 0.001 | 0.180 | 3.165 | 0.002 |
|  | Glucose | 0.018 | 0.004 | 0.141 | 4.228 | 2.660E-05 |
|  | Gender | -0.080 | 0.018 | -0.194 | -4.464 | 9.341E-06 |
|  | Height | -0.004 | 0.001 | -0.166 | -4.174 | 3.360E-05 |
|  | AC | 0.001 | 2.363E-04 | 0.081 | 2.204 | 0.028 |
|  | Pulse pressure | -0.001 | 0.001 | -0.112 | -2.008 | 0.045 |
| ApoA1/ApoB | WC | -0.009 | 0.003 | -0.139 | -2.937 | 0.003 |
|  | BMI | -0.025 | 0.007 | -0.166 | -3.600 | 3.399E-04 |
|  | Glucose | -0.030 | 0.011 | -0.093 | -2.700 | 0.007 |
|  | DBP | -0.004 | 0.001 | -0.099 | -2.765 | 0.006 |
|  | AC | 0.002 | 0.001 | 0.138 | 3.585 | 3.592E-04 |
|  | Gender | 0.220 | 0.047 | 0.209 | 4.671 | 3.564E-06 |
|  | CS | 0.008 | 0.002 | 0.136 | 3.233 | 0.001 |

TC, total cholesterol; TG, triglyceride; HDL-C, high-density lipoprotein cholesterol; LDL-C, low-density lipoprotein cholesterol; ApoA1, apolipoprotein A1; ApoB, apolipoprotein B; ApoA1/ApoB, the ratio of apolipoprotein A1 to apolipoprotein B; WC, waist circumference; CS, cigarette smoking; AC, alcohol consumption; SBP, systolic blood pressure; DBP, diastolic blood pressure; BMI, body mass index; B and Std. error, unstandardized coefficients; Beta, standardized coefficient.

**Supplementary Table 2** Relationship between serum lipid parameters and relative factors in the Maonan and Han populations without ethnic independent variable

| **Lipid** | **Risk factor** | **B** | **Std. error** | ***Beta*** | ***t*** | ***P*** |
| --- | --- | --- | --- | --- | --- | --- |
| TC | WC | 0.015 | 0.004 | 0.124 | 3.512 | 4.581E-04 |
|  | Age | 0.012 | 0.002 | 0.175 | 6.731 | 2.399E-11 |
|  | BMI | 0.033 | 0.011 | 0.105 | 2.982 | 0.003 |
|  | DBP | 0.004 | 0.002 | 0.056 | 2.105 | 0.035 |
| TG | WC | 0.044 | 0.007 | 0.229 | 6.056 | 1.771E-09 |
|  | AC | 0.005 | 0.001 | 0.107 | 4.001 | 6.615E-05 |
|  | CS | 0.016 | 0.005 | 0.087 | 3.193 | 0.001 |
|  | Glucose | 0.099 | 0.028 | 0.085 | 3.489 | 4.992E-04 |
|  | Weight | 0.017 | 0.007 | 0.101 | 2.616 | 0.009 |
| HDL-C | WC | -0.013 | 0.001 | -0.241 | -9.270 | 6.379E-20 |
|  | AC | 0.002 | 3.993E-04 | 0.160 | 5.748 | 1.094E-08 |
|  | Gender | 0.088 | 0.028 | 0.090 | 3.117 | 0.002 |
|  | Pulse pressure | -0.003 | 0.001 | -0.101 | -3.554 | 3.917E-04 |
|  | Age | 0.003 | 0.001 | 0.082 | 2.925 | 0.003 |
| LDL-C | Age | 0.012 | 0.001 | 0.214 | 8.482 | 5.287E-17 |
|  | BMI | 0.031 | 0.009 | 0.120 | 3.398 | 6.979E-04 |
|  | AC | -0.002 | 0.001 | -0.096 | -3.816 | 1.412E-04 |
|  | WC | 0.007 | 0.003 | 0.072 | 2.006 | 0.045 |
| ApoA1 | AC | 0.002 | 2.682E-04 | 0.175 | 6.216 | 6.613E-10 |
|  | WC | -0.005 | 0.001 | -0.148 | -5.751 | 1.077E-08 |
|  | CS | 0.003 | 0.001 | 0.083 | 2.936 | 0.003 |
| ApoB | WC | 0.006 | 0.001 | 0.240 | 7.060 | 2.555E-12 |
|  | Age | 0.002 | 3.342E-04 | 0.155 | 6.105 | 1.311E-09 |
|  | BMI | 0.008 | 0.002 | 0.123 | 3.653 | 2.685E-04 |
|  | DBP | 0.001 | 3.946E-04 | 0.064 | 2.528 | 0.012 |
|  | Glucose | 0.008 | 0.003 | 0.055 | 2.260 | 0.024 |
| ApoA1/ApoB | WC | -0.014 | 0.002 | -0.217 | -6.058 | 1.743E-09 |
|  | AC | 0.002 | 4.805E-04 | 0.134 | 4.764 | 2.085E-06 |
|  | Age | -0.004 | 0.001 | -0.112 | -4.460 | 8.808E-06 |
|  | BMI | -0.019 | 0.006 | -0.106 | -3.066 | 0.002 |
|  | Glucose | -0.023 | 0.010 | -0.057 | -2.288 | 0.022 |
|  | CS | 0.006 | 0.002 | 0.090 | 3.014 | 0.003 |
|  | Gender | 0.092 | 0.036 | 0.079 | 2.559 | 0.011 |

TC, total cholesterol; TG, triglyceride; HDL-C, high-density lipoprotein cholesterol; LDL-C, low-density lipoprotein cholesterol; ApoA1, apolipoprotein A1; ApoB, apolipoprotein B; ApoA1/ApoB, the ratio of apolipoprotein A1 to apolipoprotein B; WC, waist circumference; CS, cigarette smoking; AC, alcohol consumption; SBP, systolic blood pressure; DBP, diastolic blood pressure; BMI, body mass index; B and Std.error, unstandardized coefficients; Beta, standardized coefficient.

**Supplementary Table** 3 The frequencies of the two SNP alleles in different population

| Population | rs501120 alleles (%) | | rs1746048 alleles (%) | |
| --- | --- | --- | --- | --- |
|  | A | G | T | C |
| CEU | 86.9 | 13.1 | 85.5 | 14.5 |
| CHB | 62.6 | 37.4 | 68.9 | 31.1 |
| JPT | 64.9 | 35.1 | 68.6 | 31.4 |

CEU: Northern and Western Europe; CHB: Han Chinese in Beijing; JPT: Japanese in Tokyo, Japan. Collecting from the International 1000 Genomes database.

**Supplementary Table 4**. The sequences of primers, restriction enzymes for genotyping of the *CXCL12* rs501120 and rs1746048 SNPs

| SNP | Primer sequence (5’-3’) | PCR product | Restriction enzyme |
| --- | --- | --- | --- |
| rs5011120 | F: 5′-TGGTGATGGAACTGCTCTGT-3′ | 521 bp | *Hpy188*I |
|  | R: 5′-CTCCTCCTGCAGACTCACTC-3′ |  |  |
| rs1746048 | F: 5′-TGTGCCAGTTTATAGCCCCA-3′ | 257 bp | *Mwo*I |
|  | R: 5′-TCCCTTCTGTCATGGTAGCT-3′ |  |  |

SNP: single nucleotide polymorphism; F, forward pair; R, reversed pair; *Hpy188*I purchased from New England Biolabs Inc.; *Mwo*I purchased from Thermo Fisher Scientific Inc. These primers were designed and produced by Sangon, Shanghai, People’s Republic of China.

**Supplementary Table 5**. The compositions of PCR for the *CXCL12* rs501120 and rs1746048 SNPs

| SNP | Compositions of PCR | Volume/mass |
| --- | --- | --- |
| rs501120/rs1746048 | Genomic DNA | 100.0 ng |
|  | 2 × *Taq* PCR Master mix | 12.5 μL |
|  | The forward primer | 1.0 μL |
|  | The reversed primer | 1.0 μL |
|  | DNase/RNase-free water | 8.5 μL |
| Total |  | 25.0μL |

SNP: single nucleotide polymorphism; 2 × *Taq* PCR Master mix composed of 0.1 U *Taq* polymerase/μL, 500 μM dNTP each and PCR buffer

**Supplementary Table 6**. Compositions of restriction enzyme digestion for the *CXCL12* rs501120 and rs1746048 SNPs

| Compositions of restriction enzyme digestion | Volume |
| --- | --- |
| PCR product | 6.0 μL |
| Restriction enzyme (*Hpy188*I or *Mwo*I) | 0.2 μL |
| DNase/RNase-free | 8.8 μL |
| 10 × buffer solution | 1.0 μL |
| Total | 15.0 μL |

SNP: single nucleotide polymorphism; *Hpy188*I for rs501120 purchased from New England Biolabs Inc. and *Mwo*I for rs1746048 purchased from Thermo Fisher Scientific Inc.
